# Assessment of US Federal Funding of Incarceration-Related Research, 1985 to 2022

**DOI:** 10.1001/jamanetworkopen.2023.0803

**Published:** 2023-02-27

**Authors:** Samantha J. Boch, Aaron W. Murnan, Jordan F. Pollard, Nichole L. Nidey, Rose Y. Hardy, Iheoma U. Iruka

**Affiliations:** 1 College of Nursing, University of Cincinnati, Cincinnati, Ohio; 2 The James M. Anderson Center for Health Systems Excellence, Cincinnati Children’s Hospital Medical Center, Cincinnati, Ohio; 3 School of Psychology, University of Cincinnati, Cincinnati, Ohio; 4 Department of Pediatrics, Cincinnati Children’s Hospital Medical Center, University of Cincinnati, Cincinnati, Ohio; 5 Division of Biostatistics and Epidemiology, Cincinnati Children’s Hospital Medical Center, Cincinnati, Ohio; 6 Center for Child Health Equity and Outcomes Research, Nationwide Children’s Hospital, Columbus, Ohio; 7 Department of Public Policy, University of North Carolina at Chapel Hill

## Abstract

**Importance:**

The US leads the world in the raw number of incarcerated persons as well as the rate of incarceration, with detrimental effects on individual-, family-, community-, and population-level health; as such, federal research has a critical role in documenting and addressing the health-related impacts of the US criminal legal system. How often incarceration-related research is funded at the National Institutes of Health (NIH), National Science Foundation (NSF), and US Department of Justice (DOJ) levels has a direct association with the public attention given to mass incarceration as well as the efficacy of strategies to mitigate negative effects and poor health related to incarceration.

**Objective:**

To understand how many incarceration-related projects have been funded at the NIH, NSF, and DOJ.

**Design, Setting, and Participants:**

This cross-sectional study used public historical project archives to search for relevant incarceration-related keywords (eg, *incarceration*, *prison*, *parole*) since January 1, 1985 (NIH and NSF), and since January 1, 2008 (DOJ). Quotations and Boolean operator logic were used. All searches and counts were conducted and double verified by 2 coauthors between December 12 and 17, 2022.

**Main Outcomes and Measures:**

Number and prevalence of funded projects related to incarceration and prison keywords.

**Results:**

The term *incarceration* resulted in 3540 of 3 234 159 total project awards (0.11%) and *prisoner*-related terms resulted in 11 455 total project awards (0.35%) across the 3 federal agencies since 1985. Nearly a tenth of all projects funded at NIH since 1985 related to *education* (256 584 [9.62%]) compared with only 3373 projects (0.13%) that related to *criminal legal* or *criminal justice* or *correctional system* and 18 projects (0.0007%) that related to *incarcerated parents*. Only 1857 (0.07%) of all NIH-funded projects have been funded related to *racism* since 1985.

**Conclusions and Relevance:**

This cross-sectional study found that a very low number of projects about incarceration have historically been funded at the NIH, DOJ, and NSF. These findings reflect a dearth of federally funded studies investigating the effects of mass incarceration or intervention strategies to mitigate adverse effects. Given the consequences of the criminal legal system, it is undoubtedly time for researchers, and our nation, to invest more resources into studying whether this system should be maintained, the intergenerational effects of mass incarceration, and strategies to best mitigate its impact on public health.

## Key Points

**Question:**

How often have incarceration-related projects been funded at the National Institutes of Health (NIH), US Department of Justice (DOJ), and National Science Foundation (NSF)?

**Findings:**

This cross-sectional study found that a very low number of projects about incarceration have historically been funded at the NIH, DOJ, and NSF.

**Meaning:**

These findings suggest that federal funding for incarceration-related health research is inadequate, considering the size, churn, and socioeconomic consequences of the US criminal legal system.

## Teaser

This cross-sectional study compares the number of incarceration-related projects funded at the National Institutes of Health, US Department of Justice, and National Science Foundation compared with other social and behavioral risk–related projects.

## Introduction

The US spends more on health^1^ and safety^2^ than any other country, yet has some of the worst health outcomes (eg, chronic conditions, suicide, infant mortality, maternal mortality, and life expectancy).^3,4,5^ The US also incarcerates the most individuals in the world.^6^ The criminal legal system, similar to the health care system, is expansive, complex, costly, and rife with racial and economic disparities. These 2 systems, heralded and studied as separate entities, have overlap, often caring for the same populations and relying on the same agencies to transport and safeguard (eg, police, emergency medical services, social services). Given the sheer size of the US criminal legal system coupled with the undeniable link between incarceration and health inequity,^7^ addressing and understanding the economic, social, and health disparities that result from and are exacerbated by the criminal legal system are sorely needed.

In 2016, nearly 6.6 million adults in the US were under correctional supervision (approximately 2.5%), meaning incarcerated (in jail or prison), paroled, or on probation.^8^ Although US incarceration rates are slowly declining, 600 000 people are sentenced to prison and 4.9 million others cycle through jail every year.^9^ As a result, about one-third of the US adult population has a record of crime.^9^ In turn, about 1 in every 7 US adults have had an immediate family member spend at least 1 year incarcerated,^10^ and about 1 in every 14 US children have had a parent incarcerated.^7^

Importantly, there are well-documented racial and economic disparities at every level in the US criminal legal system regarding who is more likely to live in a community that is hyperpoliced^11^ and who is more likely to be stopped by police,^12,13^ arrested, sentenced,^14^ and incarcerated.^9,15,16^ Compared with 1 in every 17 White men, 1 in every 3 Black men and 1 in every 6 Hispanic men in the US are expected to go to prison during their lifetime.^17^ Black women in the US have been imprisoned at higher rates than their Hispanic and White counterparts for the past 20 years.^15^ In turn, approximately 13% of all Black or African American youths and 20% of all American Indian youths reported having lived with a parent or guardian who served time in jail or prison at some time compared with only 6% of non-Hispanic White youths.^18^ Unfortunately, these are conservative estimates due to the stigma of disclosing such information.

Criminal legal involvement is one of a few factors that legitimizes discrimination and economic sanctions in the US. Individuals with records can be denied employment, government assistance, housing, and the right to vote. These barriers doubly extend to their children. Not surprisingly, parental incarceration is considered an adverse childhood experience^19^ that is associated with negative effects on children’s mental health^20^ and educational performance,^21^ as well as increased risk for substance use and juvenile incarceration. While literature exists on the poor health and well-being of children raised in single-parent homes (compared with 2-parent households), in nonparental care,^22^ in household chaos,^23^ and in poverty, few pediatric studies acknowledge or conceptualize mass incarceration or parental involvement with the criminal legal system as an important structural and family adversity driving and exacerbating US health disparities. Taken together, the social fallout of incarceration affects individuals and their families, especially those with young children. The consequences stemming from mass incarceration must be addressed,^24^ and without research, it is unclear how to move forward.

The purpose of this study was to understand and compare the number of incarceration-related projects funded at the National Institutes of Health (NIH), the National Science Foundation (NSF), and the US Department of Justice (DOJ) compared with other social and behavioral risk–related projects at the NIH. To our knowledge, only 1 other study has sought to describe NIH funding for research examining health and health care needs of incarcerated populations.^25^ It found that less than 0.1% of all NIH grants (from 2008 through 2012) focused on criminal legal health research and less than 1.5% of the health disparities budget for 2012 was spent on criminal legal health research.^25^ We expand that study in 4 ways: (1) broadening the incarceration-related keywords to include other types of correctional-involved populations (eg, children affected by parental incarceration), (2) searching funded projects since 1985, (3) including results from the NSF and DOJ, and (4) comparing results with those of keywords related to other risk factors and social and behavioral determinants of health.

## Methods

### Study Design

This cross-sectional study was conducted to understand how many incarceration-related projects have been funded at the NIH, NSF, and DOJ. The University of Cincinnati’s Institutional Review Board waived the need for approval or informed consent because the study was conducted using publicly available data. The study followed the Strengthening the Reporting of Observational Studies in Epidemiology (STROBE) reporting guideline.

### Setting and Data Sources

We searched for all relevant keywords using free publicly available historical project archives for the NIH (NIH RePORTER, since January 1, 1985), DOJ (USAspending.gov, since January 1, 2008), and NSF (NSF Advanced Award Search via NSF.gov, since January 1, 1985). All of the public project award archive searches and total counts were conducted and double-verified by at least 2 coauthors (S.J.B. and J.F.P.) between December 12 and 17, 2022.

### Keywords of Interest

Incarceration-related keywords included the 4 primary types of detainment (*prison*, *jail*, *parole*, and *probation*), common vernacular used to describe incarcerated populations (eg, *prisoner*, *criminal*), and the stages of the US criminal legal system (eg, *arrest*, *incarceration*, *reentry*).^25^ The research team also identified common health-related risk factors and keywords related to the social and behavioral determinants of health (eg, *violence*, *health disparities*, *stress*, *trauma*, *homelessness*, *poverty*, *mental illness*)^26,27^ to compare with the results related to incarceration keywords. Quotations and Boolean operator logic were used per the NIH, NSF, and DOJ portal instructions.

### Statistical Analysis

Descriptive statistics were used to describe the total number of projects funded and the prevalence of these projects by keyword. We used Excel, version X (Microsoft Corporation) for the analysis.

## Results

Table 1 summarizes the number of total projects and awards that include incarceration-related terms in the title, abstract, or summary statement across the 3 federal agencies. The term *incarceration* resulted in 3540 total project awards (of 3 234 159 total projects [0.11%]) across the 3 federal agencies. The NIH funded the highest number of awards with *incarceration* (3223 [0.12%]) compared with the 2 other agencies (158 at DOJ [0.44%], and 159 at NSF [0.03%]). Justice-related projects were funded most, with 13 998 projects (0.43%) across the 3 federal agencies since 1985. Prisoner-related keywords resulted in 11 455 total project awards (0.35%). Terms related to incarcerated parents or incarcerated mother or father resulted in a total of 96 projects (0.003%) across the 3 federal agencies. Of the 4 primary types of detainment in the US, jail-related projects were funded most at the NIH (1765 projects), and prison-related projects were funded most at the DOJ (337 projects [0.93%]) and the NSF (215 projects [0.04%]). The NIH funded 3373 projects (0.13%) related to criminal legal or criminal justice or correctional system and 18 projects (0.0007%) related to incarcerated parents.

**Table 1.** Number of Projects That Include Incarceration Keywords in the Title, Abstract, or Summary Funded at the DOJ Since 2008 and at the NIH and NSF Since 1985

Incarceration keywords searched	Funded projects, No. (%)			
	DOJ (n = 36 171)^a^	NIH (n = 2 667 827)	NSF (n = 530 161)	Total (N = 3 234 159)
*Justice*	5641 (15.60)	6780 (0.25)	1577 (0.30)	13 998 (0.43)
*Crime*	3585 (9.91)	4386 (0.16)	785 (0.15)	8756 (0.27)
*Prisoner* or *inmate* or *criminal* or *detainee* or *perpetrator*	3667 (10.14)	6542 (0.25)	1246 (0.24)	8455 (0.26)
*Criminal legal* or *criminal justice* or *correctional system*	2876 (7.95)	3373 (0.13)	599 (0.11)	6848 (0.21)
*Victim*	5066 (14.01)	1430 (0.05)	236 (0.04)	6732 (0.21)
*Police*	2725 (7.53)	2187 (0.08)	538 (0.10)	5450 (0.17)
*Recidivism* or *reentry* or *re-entry*	685 (1.89)	3906 (0.15)	285 (0.05)	4876 (0.15)
*Incarceration* or *justice-involved* or *incarcerated*	421 (1.16)	3846 (0.14)	275 (0.05)	4542 (0.14)
*Arrested* or *legal charges* or *sentenced*	26 (0.07)	3845 (0.14)	204 (0.04)	4075 (0.13)
*Guns* or *firearms* or *weapons*	25 (0.07)	3510 (0.13)	436 (0.08)	3971 (0.12)
*Incarceration*	158 (0.44)	3223 (0.12)	159 (0.03)	3540 (0.11)
*Prison*	337 (0.93)	1509 (0.06)	215 (0.04)	2061 (0.06)
*Jail*	230 (0.64)	1765 (0.07)	30 (0.01)	2025 (0.06)
*Juvenile justice* or *detained youth* or *juvenile corrections* or *juvenile detention*	458 (1.27)	966 (0.04)	15 (0.003)	1439 (0.04)
*Probation*	310 (0.86)	890 (0.03)	40 (0.01)	1230 (0.04)
*Detention* or *detained*	136 (0.38)	479 (0.02)	104 (0.02)	719 (0.02)
*Parole*	155 (0.43)	453 (0.02)	37 (0.01)	645 (0.02)
Justice-*involved* or *justice involved*	179 (0.49)	430 (0.02)	12 (0.002)	621 (0.02)
*Incarcerated parents* or *parental incarceration* or *incarcerated father* or *incarcerated mother*	6 (0.02)	75 (0.003)	15 (0.003)	96 (0.003)
*Mass incarceration*	6 (0.02)	31 (0.002)	27 (0.005)	64 (0.002)
*School-to-prison pipeline* or *school to prison*	16 (0.04)	4 (0.0001)	6 (0.01)	26 (0.001)
*Incarcerated parents*	0	18 (0.0007)	5 (0.001)	23 (0.001)
*Children of incarcerated parents* or *youth of incarcerated parents* or *children of justice-involved parents*	3 (0.008)	15 (0.0006)	3 (0.001)	21 (0.001)

Abbreviations: DOJ, Department of Justice; NIH, National Institutes of Health; NSF, National Science Foundation.

^a^ The DOJ was limited to grants (only) vs contracts, loans, and direct payments. Search was performed December 12 to 17, 2022.

Table 2 includes the number of total projects that include common health-related risk factors and adversity keywords related to the social and behavioral determinants of health ever funded at the NIH since 1985. Among keywords searched, projects related to education or school were funded most, with approximately 256 584 projects funded or 9.62% of all NIH projects awarded since 1985. Children- or youth-related projects were second highest, with 224 869 projects funded since 1985 (8.43%). Poverty- or low-income–related projects resulted in 28 165 projects (1.06%), *stigma* or *bias* keywords resulted in 24 140 projects (0.90%), and *racism* as a keyword resulted in 1857 projects (0.07%). Homelessness-related projects resulted in 4112 projects (0.16%), while food insecurity projects resulted in 3881 projects (0.15%), and pregnancy-related projects resulted in 74 804 projects (2.78%).

**Table 2.** Number of Total Projects and Awards That Include Social Risk and Adversity Keywords in the Title, Abstract, or Summary Funded at the National Institutes of Health Since 1985^a^

Keyword	Total projects, No. (%) (n = 2 667 827)
*Education* or *school*	256 584 (9.62)
*Children* or *youth*	224 869 (8.43)
*Stress*	209 223 (7.84)
*HIV* or *AIDS*	187 359 (7.02)
*Assault* or *neglect* or *abuse*	124 516 (4.67)
*Depression* or *anxiety* or *externalizing behaviors* or *internalizing behaviors*	105 811 (3.97)
*Disability* or *disabled*	101 201 (3.79)
*Minority*	100 379 (3.76)
*Newborn* or *infant* or *neonate*	94 743 (3.55)
*Mental health* or *mental illness*	94 164 (3.53)
*White* or *Caucasian*	90 177 (3.38)
*Alcohol*	88 135 (3.30)
*Adolescents* or *adolescence*	82 265 (3.08)
*Substance used* or *illegal drug use* or *addiction*	75 293 (2.82)
*Pregnant* or *pregnancy*	74 084 (2.78)
*Black* or *African American*	71 915 (2.70)
*Trauma*	55 270 (2.07)
*Bipolar* or *schizophrenia* or *psychosis* or *psychotic* or *delusion disorder*	50 033 (1.88)
*Latina* or *Latino* or *Latinx* or *Hispanic*	42 206 (1.58)
*Health disparities* or *health inequities*	34 361 (1.29)
*Opioid* or *opiate*	33 587 (1.26)
*Poverty* or *low-income* or *low income* or *impoverished*	28 165 (1.06)
*Social support* or *social connectedness* or *social connection*	26 562 (1.06)
*Stigma* or *bias*	24 140 (1.09)
*Suicide* or *suicidal*	15 851 (0.59)
*Veteran* or *military* or *army* or *navy*	15 391 (0.58)
*American Indian* or *Native American* or *Indigenous*	14 789 (0.55)
*Housing instability* or *homelessness* or *homeless*	4112 (0.15)
*Sexually transmitted infections* or *sexually transmitted diseases*	12 576 (0.47)
*Poverty*	12 416 (0.47)
*Asian* or *Pacific Islander*	10 999 (0.42)
*Immigrant* or *immigration* or *refugee*	9674 (0.36)
*Marijuana* or *weed* or *cannabis*	8.017 (0.30)
*Transportation*	6783 (0.25)
*Heroin*	6289 (0.24)
*Gay* or *lesbian* or *bisexual* or *transgender* or *transsexual* or *asexual* or *intersex* or *LGBT* or *two spirit* or *two-spirit*	6127 (0.23)
*Toxic stress* or *chronic stress*	5984 (0.22)
*Childcare* or *child care* or *day care*	5922 (0.22)
*Health equality* or *health equity*	4979 (0.19)
*Social determinants* or *social risk* or *social ills*	4064 (0.15)
*Food insecurity* or *hunger* or *food instability*	3881 (0.15)
*Politics* or *political*	3374 (0.13)
*Heterosexual*	3136 (0.12)
*Foster care* or *child welfare* or *foster agency*	2865 (0.11)
*Misconduct* or *conduct disorder*	2759 (0.10)
*Rape* or *sexual assault*	2163 (0.08)
*Interpersonal violence* or *domestic violence*	2231 (0.08)
*Jewish* or *Jew*	2666 (0.10)
*Interpersonal violence* or *domestic violence*	2231 (0.08)
*Racism*	1857 (0.07)
*Harm reduction*	1667 (0.06)
*Sex work* or *sex workers* or *prostitute* or *sex trafficking* or *human trafficking*	1616 (0.06)
*Adverse childhood experiences* or *ACEs* or *childhood adversity*	1629 (0.06)
*Homicide* or *murder*	1073 (0.04)
*Residential treatment*	795 (0.03)
*Intersectionality*	661 (0.02)
*Neonatal abstinence syndrome*	535 (0.02)
*Truancy*	134 (0.01)
*Positive childhood experiences* or *PCEs* or *counter ACEs* or *BCEs*	34 (0.001)
*White supremacy* or *White privilege*	1 (0.00004)

Abbreviations: ACE, adverse childhood experience; BCE, benevolent childhood experience; PCE, positive childhood experience.

^a^ Search was performed from December 12 to 17, 2022.

## Discussion

The findings of this cross-sectional study reflect a lack of federally funded studies investigating the effects of mass incarceration or intervention strategies to mitigate these potential adverse effects. Consistent with previous research,^25^ 0.12% of all projects funded at the NIH and 0.03% at the NSF since 1985 were related to incarceration. This is substantially lower than the number of projects that relate to other systems such as education (243 989 projects [9.38%]) and the military (15 931 projects [0.58%]). Considering the size, churn, and socioeconomic consequences of the US criminal legal system, this is inadequate and unacceptable. Evidence in the current study provides a snapshot of how little attention the top federal research agencies have given to criminal legal research and interventions. Additional research is needed to better understand how to improve the health, services, and systems that care for people and communities affected by incarceration at all stages (upon arrest, during incarceration, and upon reentry) and across generations.

Some may argue that examining mass incarceration as a possible hazardous exposure for individuals and their families may be outside the scope or too large of an issue to address within the health care system. Reports issued by the National Academies of Sciences, Engineering and Medicine^28,29^ and other scholars^30^ in support of reducing adverse childhood experiences say otherwise. We need to understand the association of intergenerational transmission of stress and trauma, especially due to the incarceration of parents, and children’s well-being, so we can develop appropriate interventions. Methodologically, it is challenging to disentangle the effects of large institutions such as mass incarceration because they are largely influenced by deeper rooted systemic factors that underpin American culture (such as capitalism, colonialism, and racism). Unfortunately, our findings also demonstrate that very few projects related to racism have been investigated at the NIH (1857 projects [0.07%]). This begs the question that Davis asked 20 years ago in her book, *Are Prisons Obsolete?*^31^: “Is racism so deeply entrenched in the institution of the prison that it is not possible to eliminate one without eliminating the other?” Alternatively, it could be argued that incarceration-related funding disparities are reflective of extra safeguards and protections afforded to incarcerated populations following extensive and long-standing maltreatment of incarcerated persons within US history. However, if this were true, we would also observe similarly low rates of funded research on children and youth (224 869 projects [8.43%]) and pregnancy (74 084 [2.78%]), which is not the case. Better inclusion, conceptualization, and discussion of the role the criminal legal system has had on the health of the US is needed in research, especially in research that investigates or attempts to mitigate social ills such as racism, substance use, mental illness, poverty, homelessness, violence, and childhood adversity. Continuation and funding of this type of research without inclusion or discussion of the role the criminal legal system has had will only maintain or widen health disparities. We hypothesize that the US will continue to lead in both incarceration and poor health outcomes until we acknowledge, investigate, and mitigate the interplay between health and safety.

Funded research largely dictates what we know, what our evidence base is, and what health-related factors our nation prioritizes. The lack of federal focus on such a critical health-related factor associated with population health represents, at best, an oversight, and at worst, a reflection of the proximity to the same structures that have created and upheld the criminal legal system in the US (eg, structural racism). As such, the lack of funded projects focusing on the health fallout for our incarcerated population may very well have contributed to the criminal legal system status quo. A lack of research in this area may also reflect a lack of concern, awareness, and/or training among federal investigators and funders, which is more malleable and readily targetable. Criminal legal system– and incarceration-related training could be included in health professional programs and related curriculum. Strategic plans and funding opportunities of the federal agencies and institutes could start to include the criminal legal system as an important structural inequity to investigate and mitigate. According to the strategic plans of all the NIH institutes, only 2 explicitly mention justice system settings (the National Institute of Nursing Research and National Institute of Drug Abuse). However, the National Institute on Minority Health and Health Disparities, the National Institute of Mental Health, the National Institute of Child Health and Development, the National Institute on Alcohol Abuse and Alcoholism, and the National Institute on Aging (for aging incarcerated populations) could all directly incorporate this as a priority area and setting. Immediate actions and recommendations to increase NIH support have been described^25^ and could include an increase to both NSF and DOJ support (eg, adding incarceration-related questions to existing nationally representative health data sets, increasing funding, including guidelines on safe and ethical conduct of criminal justice health research to reduce barriers, establishing training and career development awards focused on correctional health settings, highlighting existing efforts and innovation). In addition, alleviating the racial disparities in who is funded by federal agencies^32,33^ by funding additional scholars from racial and ethnic minority groups or from disadvantaged communities, including those with histories of personal or familial incarceration may also be important.

### Limitations

This study has some limitations. We were unable to wholly verify the accuracy of the public databases in aggregating the total number of projects related to incarceration and other social risk keywords, and our results may underestimate the true number of projects funded at these agencies historically. For instance, the term *incarcerated* is also used in the biomedical term *incarcerated hernia*, and *juvenile* is often used to describe *juvenile diabetes*. Despite these instances, this study creates a novel contribution to the sciences and likely underestimates the criminal legal system–related funded projects.

## Conclusions

Given the high costs and collateral consequences on the health and economic standing for those incarcerated, their families, and their communities, it is undoubtedly time for researchers to invest resources into studying the intergenerational effects of mass incarceration, strategies to best mitigate its impact on public health, and whether or not we should continue to maintain such a system.^29,34,35^ The findings of this cross-sectional study suggest that academic researchers and federal agencies should prioritize funding and training related to the socioeconomic effects of the criminal legal system. Careful examination of the net public health benefits and costs of such a massive system is sorely needed to understand how to better care for the health and well-being of persons, families, and communities affected by incarceration.

## Notes

Supplement.
Data Sharing Statement
