# Supplementary material for: Assessment of US Federal Funding of Incarceration-Related Research, 1985 to 2022
Source: JAMA Netw Open. 2023 Feb 27;6(2):e230803. doi: 10.1001/jamanetworkopen.2023.0803 (PMC9972190; doi:10.1001/jamanetworkopen.2023.0803)
Supplement: Supplement. — Data Sharing Statement [file jamanetwopen-e230803-s001.pdf]

Data Sharing Statement

Boch. Assessment of US Federal Funding of Incarceration-Related Research, 1985 to 2022. *JAMA Netw Open*. Published February 27, 2023. doi:10.1001/jamanetworkopen.2023.0803

Data
Data available: No

Additional Information
Explanation for why data not available: Data are already publicly available.
